# Genome‐wide association mapping for field spot blotch resistance in South Asian spring wheat genotypes

**DOI:** 10.1002/tpg2.20425

**Published:** 2024-01-14

**Authors:** Umesh Kamble, Xinyao He, Sudhir Navathe, Manjeet Kumar, Madhu Patial, Muhammad Rezaul Kabir, Gyanendra Singh, Gyanendra Pratap Singh, Arun Kumar Joshi, Pawan Kumar Singh

**Affiliations:** ^1^ ICAR‐Indian Institute of Wheat and Barley Research Karnal Haryana India; ^2^ International Maize and Wheat Improvement Centre (CIMMYT) Apedo Mexico City Mexico; ^3^ Agharkar Research Institute Pune India; ^4^ ICAR‐Indian Agricultural Research Institute New Delhi India; ^5^ Bangladesh Wheat and Maize Research Institute (BWMRI) Dinajpur Bangladesh; ^6^ ICAR‐National Bureau of Plant Genetic Resources New Delhi India; ^7^ Borlaug Institute for South Asia New Delhi India

## Abstract

Spot blotch caused by *Bipolaris sorokiniana* ((Sacc.) Shoemaker) (teleomorph: *Cochliobolus sativus* [Ito and Kuribayashi] Drechsler ex Dastur) is an economically important disease of warm and humid regions. The present study focused on identifying resistant genotypes and single‐nucleotide polymorphism (SNP) markers associated with spot blotch resistance in a panel of 174 bread spring wheat lines using field screening and genome‐wide association mapping strategies. Field experiments were conducted in Agua Fria, Mexico, during the 2019–2020 and 2020–2021 cropping seasons. A wide range of phenotypic variation was observed among genotypes tested during both years. Twenty SNP markers showed significant association with spot blotch resistance on 15 chromosomes, namely, 1A, 1B, 2A, 2B, 2D, 3A, 3B, 4B, 4D, 5A, 5B, 6A, 6B, 7A, and 7B. Of these, two consistently significant SNPs on 5A, TA003225‐0566 and TA003225‐1427, may represent a new resistance quantitative trait loci. Further, in the proximity of *Tsn1* on 5B, AX‐94435238 was the most stable and consistent in both years. The identified genomic regions could be deployed to develop spot blotch‐resistant genotypes, particularly in the spot blotch‐vulnerable wheat growing areas.

## Core Ideas

- Resistant germplasm identification to spot blotch of wheat.
- Single‐nucleotide polymorphism markers associated with spot blotch resistance.
- Genome‐wide association studies analysis for spot blotch resistance in Indian spring wheat panel.

**Abbreviations**
AUDPC: area under the disease progress curve
LD: linkage disequilibrium
QTL: quantitative trait loci
SB: spot blotch
SNP: single‐nucleotide polymorphism

## 1 INTRODUCTION

The importance of wheat for food security is well known historically but got enhanced attention from the Green Revolution period (Joshi et al., 2007). However, the 3Cs, that is, climate change, COVID‐19, and the rising cost of living threaten agricultural production and food security globally. Climate change is increasing global food insecurity with heat waves, excessive rainfall, droughts, etc., and subsequently, farmers may face reduced agricultural production. COVID‐19 pandemic disrupted global food supply systems and long‐term pandemic effects include economic and societal upheaval. Any climate‐related disruption to food distribution and delivery, worldwide or locally, may affect food security, quality, access, and cost of living. As per FAO et al.'s (2022) report, approximately 2.3 billion people, that is, 30% of the total global population, are moderately or severely affected by food insecurity. Climate‐resilient and higher yielding genotypes play a key role in the assurance of yield and income to farmers (Joshi et al., 2007). Therefore, more efforts are required to develop new, high‐yielding cultivars having the potential to withstand biotic and abiotic stresses.

Among biotic stresses, several disease‐causing pathogens are responsible for yield reduction in wheat. However, spot blotch (SB) caused by *Bipolaris sorokiniana* ((Sacc.) Shoemaker) (teleomorph: *Cochliobolus sativus* [Ito and Kuribayashi] Drechsler ex Dastur) is of major concern in warm wheat growing areas of the world, such as eastern India, Bangladesh, Nepal, China, Southeast Asia (Thailand, Philippines, Indonesia), Latin America (Bolivia, warmer regions of Brazil, Paraguay, northeast Argentina), and Africa (Tanzania and Zambia) (Chattopadhyay et al., 2022; Duveiller & Sharma, 2009; Juliana et al., 2022). Under hot and humid conditions, SB can cause 15%–20% production losses and may result in seed discoloration, shriveled seeds, and loss of viability (Chattopadhyay et al., 2022; Gupta et al., 2018; Singh et al., 2018). SB is estimated to affect 25 million ha of wheat globally, of which 10 million ha are under the rice–wheat cropping system in the Indian subcontinent alone (Duveiller et al., 2005; Joshi et al., 2007). In the rice–wheat cropping system, specific agronomic approaches, such as late sowing and unbalanced fertilizer application may increase disease frequency (Gupta et al., 2018; Joshi et al., 2007; Juliana et al., 2022). The SB pathogen thrives under high temperature (>26°C) and high humidity, which is why late‐sown wheat in South Asia normally has high SB infection (Gupta et al., 2018; Pandey et al., 2021). If predictions of a rise in mean temperature due to climate change prove accurate, the disease is expected to pose a threat to global wheat production (Gupta et al., 2018).

*Bipolaris sorokiniana* causes blotches on leaves, node canker, and possibly seedling blight (Chand et al., 2021; Singh et al., 2018). Dark brown lesions with a 1‐ to 2‐mm diameter and non‐chlorotic edges indicate early infection. The susceptible variety, however, sees these small leaf lesions grow into elongated light to dark brown spots that coalesce and induce necrosis of the leaf tissue (Chand et al., 2010; Duveiller et al., 2005). Just like other wheat diseases caused by hemibiotrophic fungi, host immunity to SB is yet to be discovered, and even resistant lines suffer yield losses under high disease pressure (Chand et al., 2021; Gupta et al., 2022; Singh et al., 2015).

Genetic studies suggested a quantitative genetic control for SB resistance in wheat, which is influenced by genotype‐by‐environment interaction (Chand et al., 2021; Gupta et al., 2018; Joshi et al., 2004; Juliana et al., 2022). The development of SB resistance wheat cultivars may be accelerated by using molecular markers identified in bi‐parental quantitative trait loci (QTL) mapping and genome‐wide association studies (GWASs) (Singh et al., 2018). Several QTLs associated with resistance to SB have been discovered on different chromosomes (Adhikari et al., 2012; Ahirwar et al., 2018; Bainsla et al., 2020; Gonzalez‐Hernandez et al., 2009; Gurung et al., 2014; Jamil et al., 2018; Juliana et al., 2022; Kumar et al., 2015; Lillemo et al., 2013; Lu et al., 2016; Neupane et al., 2007; Sharma et al., 2007; Singh et al., 2018; Tomar et al., 2021; Zhang et al., 2020). So far, four QTLs with major effects have been assigned the designations *Sb1* to *Sb4*. *Sb1* was assigned to chromosome 7DS based on the findings of Lillemo et al. (2013). This gene was in close proximity to the cloned leaf rust resistance gene *Lr34*, which has been shown to exhibit pleiotropic effects on yellow rust (*Yr18*), stem rust (*Sr57*), powdery mildew (*Pm38*), and leaf tip necrosis (*Ltn+*) (He et al., 2022). The other three *Sb* genes have been well mapped, that is, *Sb2* on 5B (Kumar et al., 2015), *Sb3* on 3B (Lu et al., 2016), and *Sb4* on 4BL (Zhang et al., 2020), but so far they have not been cloned.

The objectives of this research were to identify novel genomic areas linked with SB resistance and resistance donors that could be used in breeding in a panel of 174 genotypes from India, Bangladesh, Nepal, and the International Maize and Wheat Improvement Center (CIMMYT), Mexico. The panel was previously used by Phuke et al. (2020) and He et al. (2021) for studies on tan spot and wheat blast, respectively.

**Core Ideas**

- Resistant germplasm identification to spot blotch of wheat.
- Single‐nucleotide polymorphism markers associated with spot blotch resistance.
- Genome‐wide association studies analysis for spot blotch resistance in Indian spring wheat panel.

## 2 MATERIALS AND METHODS

### 2.1 Plant material and genotyping

A panel of 174 spring wheat genotypes from CIMMYT‐Mexico (97), India (30), Bangladesh (19), and Nepal (28) were used (Table S1). These genotypes represent the contemporary elite breeding lines from the respective organizations or countries.

The panel was genotyped with Illumina Infinium 20k wheat array (15k + 5k add‐on array) at Trait Genetics GmbH, Germany, where samples were analyzed on Infinium ultra‐HD chips carrying 24 samples each. The single‐nucleotide polymorphism (SNP) markers were filtered according to the following criteria: uncertain position (1695 markers), minor allele frequency less than 5% (2707 markers), and missing data points of more than 10% (222 markers), which resulted in 11,499 markers available for GWAS analysis. Marker locations on the reference whole genome sequence (IWGSC: Chinese Spring RefSeq v1.0, International Wheat Genome Sequencing 2018) were obtained from the following database: https://wheat‐urgi.versailles.inra.fr/.

### 2.2 Disease screening

Field experiments for SB were conducted in CIMMYT's Agua Fria station, Mexico, during the 2019–2020 (hereafter referred to as 2020) and 2020–2021 (referred to as 2021) cropping seasons. The experiment was conducted in a randomized complete block design with two replications. The experimental unit consisted of 1‐m double‐row plots, and four checks: Chirya‐3 (resistant), Francolin (moderately resistant), Ciano T79, and Sonalika (susceptible) were included.

The virulent local isolates of *B. sorokiniana* previously isolated and kept in freezers at −20°C were reactivated and cultured on V8 medium for 5–7 days at 22–25°C for mycelia growth. Subsequently, the isolates were multiplied on sorghum seeds previously soaked and autoclaved. *Bipolaris sorokiniana* inoculated sorghum grains were placed in flasks and then incubated for 6 weeks at room temperature with frequent shaking to mix the grains and promote better fungus coverage. The sorghum grains were then scattered at the base of the plants in the middle of the double row for field inoculation after 21 days of planting at the 3‐ to 5‐leaf stage. Four to five weeks after inoculation, disease severity was visually scored for each plot using the double‐digit scale (00–99) (Saari & Prescott, 1975). The first digit (D1) indicates disease progress in canopy height from the ground level, and the second digit (D2) refers to severity measured based on the diseased leaf area. Both D1 and D2 were scored on a scale of 1–9. The disease evaluation was repeated four times at 7‐ to 10‐day intervals. For each evaluation, the percentage of disease severity was estimated based on the following formula:

$$\%\;\mathrm{Severity}\;=\;\left(\frac{D1}{9}\right)\;\times \;\left(\frac{D2}{9}\right)\;\times \;100$$

The area under the disease progress curve (AUDPC) was calculated from the four disease evaluations using the following formula:

$$\mathrm{AUDPC}\;=\sum_{i\;=\;0}^{n\;=\;1}[\{Y_{i}+Y_{i\;+\;1})÷2\}\;\times \;\left(t_{i\;+\;1}-t_{i}\right)$$

where *Y_i_* is the SB severity at time *t_i_*, *t_i_*_+ 1_ − *t_i_* is the time interval (days) between two disease scores, and *n* is the number of times when SB was recorded.

### 2.3 Linkage disequilibrium and population structure

Using all 11,499 SNP markers, a kinship matrix and clusters of individual genotypes were calculated, whereas a heat map was generated using a classical equation (VanRanden, 2008) in R program. TASSEL 5 (http://www.maizegenetics.net) was used to determine the linkage disequilibrium (LD) parameters among the SNP markers, which were then displayed against the physical distances.

Numeric transformation of genotypic data was performed using the R package GAPIT3 (Wang & Zhang, 2021) in accordance with the required format of the Structure 2.3.4 software (Pritchard et al., 2000), in which the admixture model was used for structure analysis. The burn‐in period was set to 100,000, followed by 500,000 markers chain Monte Carlo (MCMC) replications. The subpopulation test range was retained from K1 to K10, each having five iterations (runs). The real subpopulations were assessed using the K method (Earl & VonHoldt, 2012), which was then validated by the Evanno et al. (2005) method using the STRUCTURE HARVESTER tool (Earl & VonHoldt, 2012). The standard deviation and the average logarithm of the probability of the observed likelihood (LnP[D]) were derived from the output summary. LnP(D) was calculated for each stage of the MCMC for each class (K = 1–10) by computing the log‐likelihood for the data.

A neighbor‐joining tree was created using TASSEL 5.0 (Bradbury et al., 2007) and visualized using the iTOL website (Letunic & Bork, 2021).

### 2.4 Principal component analysis

Principal component analysis (PCA) was performed using 11,499 SNPs and 174 genotypes in fixed and random model circulating probability unification (FarmCPU) (Liu et al., 2016). The first two principal components were drawn to show the clustering among genotypes. The intra‐chromosomal LD was calculated as the pairwise marker correlations (*r* ^2^) between the SNP markers plotted against the physical distance for significant marker‐trait associations (MTAs). The long‐distance LD and spline were fitted to the LD‐decay graph using *r* ^2^ values of less than 0.99 using ggplot2 v3.30 in R v3.5.2.

### 2.5 Statistics and genome‐wide association analysis

The combined analysis of variance (ANOVA) was carried out for the 2‐year experiment, and three variance components, that is, genotypic variance $\sigma^{2}$, experimental variance $\sigma_{E}^{2}$, and interaction of genotype and experiment variance ${\sigma^{2}}_{gE}$, were estimated for SB using restricted maximum likelihood (Patterson & Thompson, 1971) estimation procedure using software META‐R v. R‐3.3.1.

Broad‐sense heritability was estimated using the following formula:

$$\;H^{2}=\frac{\sigma_{g}^{2}}{\;\sigma_{g}^{2}+\frac{\sigma_{gE}^{2}}{\mathrm{nEnv}}\;+\;\frac{\sigma_{e}^{2}}{\mathrm{nEnv}\times \mathrm{nrps}}}\;$$

where $\sigma_{g}^{2}$ and $\sigma_{e}^{2}$ are the genotype and error variance, respectively, $\sigma_{gE}^{2}$ is the genotype‐by‐environment interaction variance, nEnvs is the number of environments, and nrps is the number of replications.

The Bartlet test assessed the homogeneity of error variance before pooling the two‐experiment data for GWAS analysis. MTA was performed using a mixed‐linear model (MLM) and FarmCPU. The GWAS analysis using the FarmCPU model was performed using the R software package GAPITv.3.5 (Wang et al., 2021). The GWAS study was conducted for the two experiments separately, as well as for pooled experimental data. The markers were declared significant using Bonferroni correction with significant cutoff (*p*‐values, 3.0 E‐06) calculated at the alpha level of 0.2 using 11,499 markers to reduce false discovery rate in both MLM and FarmCPU models.

In TASSEL v.5 (Bradbury et al., 2007), the MLM (Yu et al., 2006) was fitted, where population structure was used as a fixed effect and kinship was used as a random effect. Two principal components were used to account for population structure (Price et al., 2006), and kinship was obtained by the centered identity‐by‐state method (Endelman & Jannink, 2012). We ran the MLM using the optimum compression level and the “population parameters previously determined” (Zhang et al., 2010) options in TASSEL. The *p*‐values for the significance tests of the marker‐trait associations, the marker effects, and the percentage of the SB variation explained by each marker were obtained. The LD between the consistent markers was analyzed using TASSEL version 5, and the standardized disequilibrium coefficients (D0) (Lewontin, 1964), the correlations between alleles at the two marker loci (*r* ^2^), and the *p*‐values for the existence of LD using the two‐sided Fisher's exact test were obtained. Markers with high *r* ^2^ values, *D'* values, and *p*‐values for the test of disequilibrium equal to zero were grouped into LD blocks.

### 2.6 Gene functional annotations

GWAS results were further analyzed to test if the identified MTAs fall within the known genomic regions using functional annotation from the reference genome assembly (IWGSC Ref Seq v1.0). From the genome annotations provided by IWGSC, functional annotation of the genes, either containing significant SNPs or close by them, was collected and checked for their possible association with SB resistance. Protein functions were then retrieved from annotated data using literature mining. Only the genes in the exact genomic location were considered, and the number of base pairs added changed for each marker based on how close it was to the genes. The interval was then explored for predicted genes, and annotations from the IWGSC (https://www.wheatgenome.org/) were obtained. For several genes, the IWGSC annotations were not available. Thus, they were evaluated based on orthologous genes in related species with known predicted functions using the comparative genomics tool in Plant Ensembl. The *Triticum aestivum* gene transcripts and their available domains in Ensembl were also used (using the show transcript table link). Moreover, the JBrowse tool from T3/Wheat (https://wheat.triticeaetoolbox.org/; Blake et al., 2016) and GBrowse from URGI (https://urgi.versailles.inra.fr/gb2/gbrowse/wheat_survey_sequence _annotation) were also used to identify annotation to SNP markers.

### 2.7 Stacking resistance alleles

Resistance alleles were determined by comparing the mean of corrected AUDPC between alleles using the Wilcoxon test implemented in the R package “ggpubr” (Kassambara, 2020). Wheat lines were grouped by the number of resistance alleles they contained, and Tukey's honest significant difference test (*p* < 0.05) implemented in the R/multcomView package was used to compare whether there were significant differences in mean disease severities between groups.

### 2.8 Haplotype analysis

One stable SNP marker, 5B_AX‐94435238, was selected for haplotype analysis. A pairwise comparison of corrected disease severities between haplotypes was conducted using the Wilcoxon test implemented in the R package “ggpubr” (Kassambara, 2020). Corrected AUDPC values were obtained using best linear unbiased prediction analysis for 2020 and 2021 and mean (across the environments) using Meta R (Alvarado et al., 2020).

## 3 RESULTS

### 3.1 Phenotypic evaluation and heritability

**FIGURE 1:**
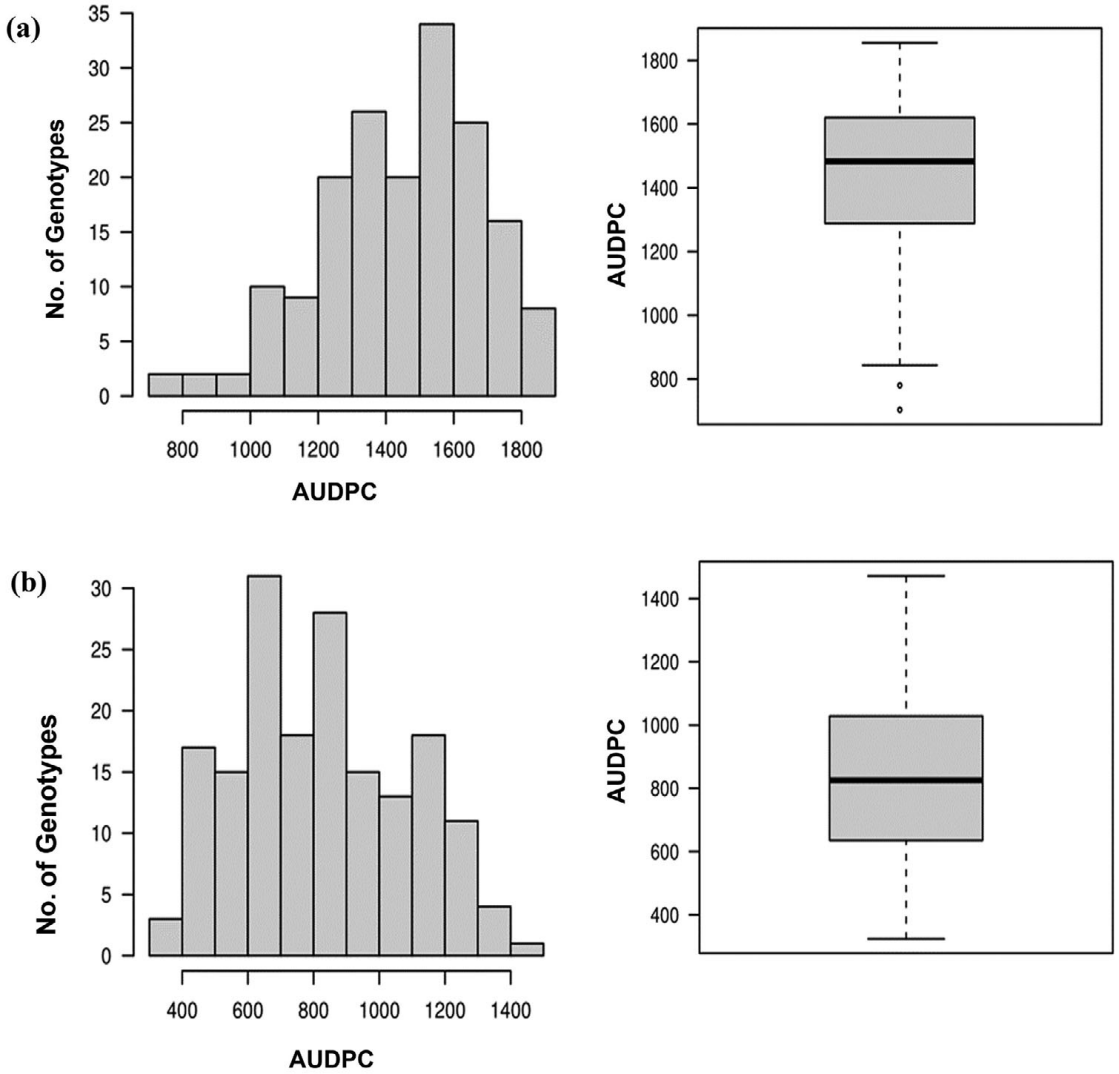
Phenotypic distribution of 174 spring wheat genotypes for area under the disease progress curve (AUDPC) in spot blotch (SB) during (a) 2020 and (b) 2021. The lines in the boxplot represent the median of the distribution, while the black dots are outliers.

The AUDPC for SB ranged from 433.38 to 1813.43 in 2020 and from 351.77 to 1435.67 in 2021, displaying considerable phenotypic variation with a continuous distribution of lines in both years (Figure 1). Among the tested entries, CIM 33 (TEPOCA T 89) was the most susceptible (AUDPC = 1644.76), while CIM 34 (MILAN) was the most resistant (AUDPC = 558.91). The AUDPC for the resistant check Chirya 3 was recorded at 756.06, while it was 1744.69 for the susceptible check Ciano T79 (Table 1). The ANOVA revealed the highest heritability in 2021 (0.94), followed by 2020 (0.93), as well as a high heritability across years (0.86). ANOVA revealed significant effects from genotype, year, and genotype‐by‐year interaction (*p* < 0.0001; Table 2).

**TABLE 1:** List of top wheat genotypes showing resistance to *B. sorokiniana* under field conditions.

Sr. No.	Entry	Pedigree	AUDPC 2020	AUDPC 2021	Mean AUDPC
1	CIM‐34	MILAN	703.4	436.42	569.91
2	CIM‐87	SW8488*2/KURUKU	843.51	324.07	583.79
3	CIM‐75	BORL14//KFA/2*KACHU	780.25	512.04	646.14
4	CIM‐39	VOROBEY	865.43	445.06	655.25
5	CIM‐69	ATTILA*2/PBW65//KACHU/3/UP2338*2/KKTS*2//YANAC	929.32	464.51	696.91
6	CIM‐83	SW91.4903/3/URES/BOW//OPATA/4/SW94.15373	985.15	423.46	704.3
7	CIM‐96	JWS 17/7/IAS58/4/KAL/BB//CJ71/3/ALD/5/CNR/6/THB/CEP7780/8/FINSI	1050.09	401.85	725.97
8	IND‐15	C 306	1094.01	365.12	729.57
9	IND‐1	HD 2967	1090.92	442.9	766.91
10	CIM‐84	MON/TAN//ROMO96/3/METSO/4/FINSI	1103.13	442.9	773.02
11	IND‐7	PBW 644	1094.01	455.86	774.94
12	IND‐33	MACS 6478	1068.03	492.59	780.31
13	CIM‐91	BAV92//IRENA/KAUZ/3/HUITES	1001.39	559.57	780.48
14	CIM‐89	FINSI/METSO	1160.65	401.85	781.25
15	IND‐16	DBW 39	1093.7	475.31	784.5
16	IND‐19	MAGHAR	1129.73	445.06	787.4
17	CIM‐46	BOKOTA	1018.21	587.65	802.93
18	CIM‐97	MILAN/ARA90//TNMU/TUI	1178.28	445.06	811.67
19	CIM‐8	MUTUS//KIRITATI/2*TRCH/3/WHEAR/KRONSTAD F2004	1075	557.41	816.2
20	CIM‐78	KASUKO	1100	557.41	828.7
	Check‐R	Chirya 3	1050.85	461.26	756.06
	Check‐S	CIANO T79	1943.53	1545.83	1744.69

*Note*: CIM indicates the germplasm originated from International Maize and Wheat Improvement Center (CIMMYT), and IND indicates the germplasm originated from India.

Abbreviation: AUDPC, area under the disease progress curve.

**TABLE 2:** Statistics and analysis of variance of the 174 advanced lines using area under the disease progress curve (AUDPC).

Statistics	2020	2021	Mean
Heritability	0.93	0.94	0.86
Genotype variance	98,523.91	62,587.63	64,933.86
Residual variance	14,791.98	7312.90	11,052.44
Grand mean	1260.51	826.00	1043.26
LSD	231.52	164.06	270.79
CV	9.65	10.35	10.08
Genotype significance	0	0	1.58E‐33
Gen × Env significance	–	–	2.96E‐26
Env significance	–	–	0.001432

Abbreviations: CV, coefficient of variation; Env, environment; Gen, genotype; LSD, least standard deviation.

### 3.2 SNP density and principal component analysis

Among polymorphic SNP markers, 40.9% (5754), 50.8% (7142), and 8.3% (1167) were from the A, B, and D genomes, respectively. With a genomic coverage of 13.9 GB and 14,063 markers across the genome, the average marker density was 1.9 Mb between markers. The lowest marker density, 7.03 Mb between markers, was at chromosome 4D, while the highest, 0.54 Mb between markers, was observed at chromosome 2B. The average distance between markers for A, B, and D genomes was 0.89, 0.84, and 3.92 Mb, respectively.

**FIGURE 2:**
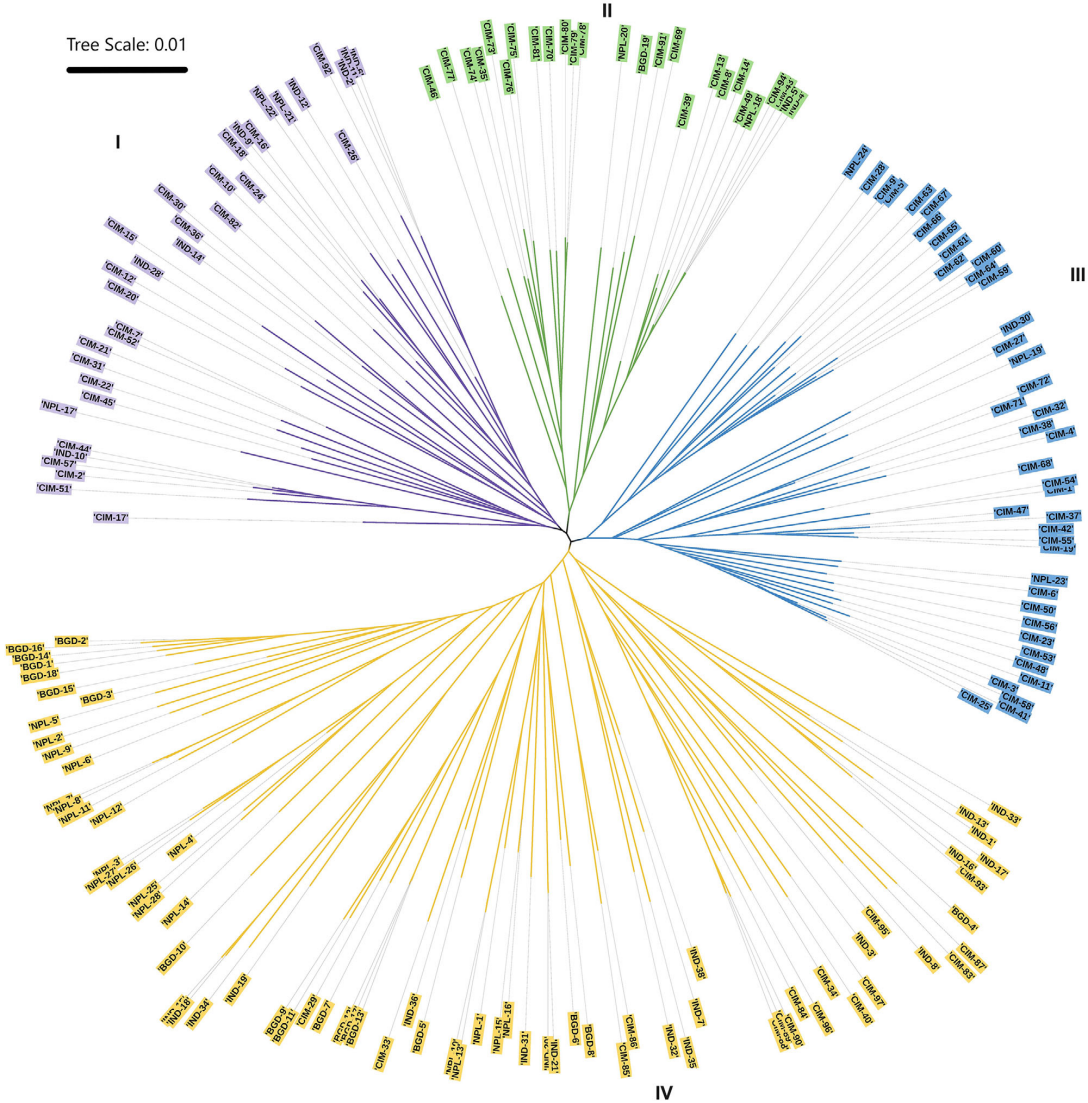
Population structure of 174 diverse spring wheat germplasms revealed by structure and neighbor joining tree.

**FIGURE 3:**
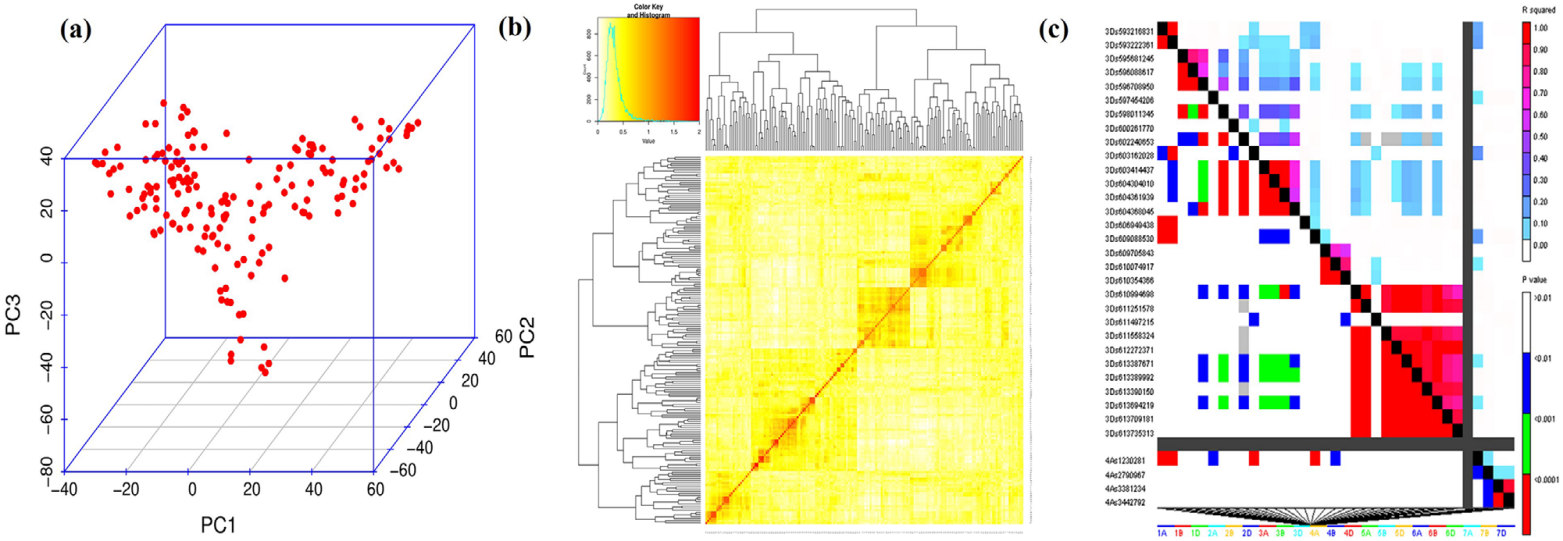
(a) Principal component analysis (PCA) of 11,499 single‐nucleotide polymorphism (SNP) markers in 174 spring wheat lines. (b) Heat map with a high kinship relationship among 174 spring wheat lines, and (c) linkage disequilibrium (LD) block obtained from TASSEL v.5 for 11,499 SNP markers.

Population structure analysis revealed four groups, of which Group 1 (G‐I) consisted of 73 lines, majorly belonging to South Asia. Group 2 (G‐II) consisted of 41 lines, Group 3 (G‐III) consisted of 34 lines, while Group 4 (G–IV) consisted of 26 lines, mostly belonging to the CIMMYT origin (Figure 2). Based on pedigree information, most lines within the group shared descendents from common parents. Principle component analysis of a similar structure was observed, as shown in Figure 3. The heat map showed a high kinship relationship among lines (Figure 3).

### 3.3 Marker‐trait associations on SB resistance

**FIGURE 4:**
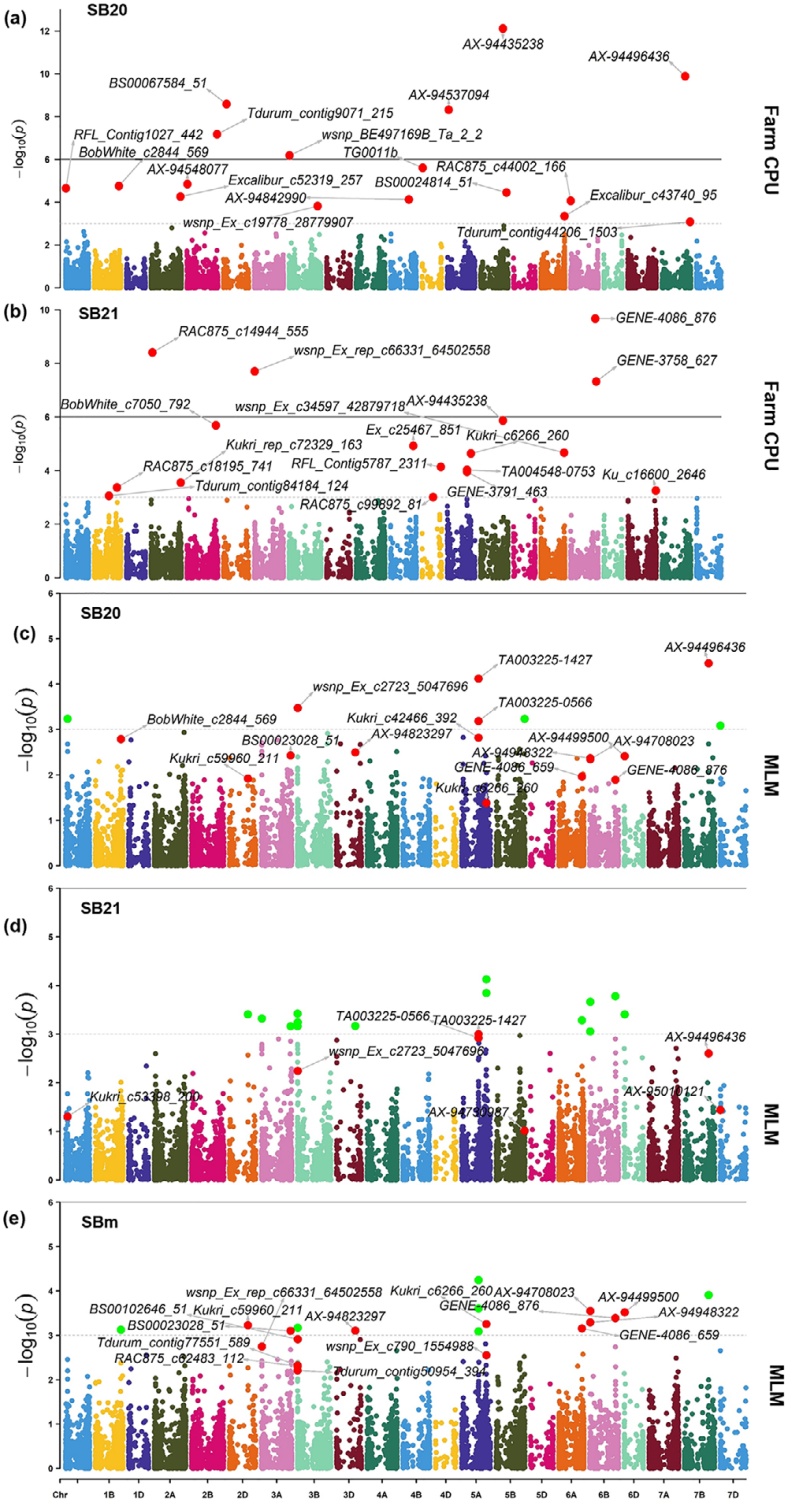
Manhattan plots representing 21 chromosomes (a–b) show the significant markers detected by fixed and random model circulating probability unification (FarmCPU) model for spot blotch (SB) during 2020 and 2021, while (c–e) show the significant markers detected by mixed‐linear model (MLM) model for SB during 2020, 2021 and mean area under the disease progress curve (AUDPC).

A total of 20 SNP markers (MLM and FarmCPU models) showed significant association with SB resistance, spreading over 15 chromosomes, namely, 1A, 1B, 2A, 2B, 2D, 3A, 3B, 4B, 4D, 5A, 5B, 6A, 6B, 7A, and 7B. The significant SNP “5B_AX‐94435238” identified with the FarmCPU model was the most stable and consistent in both years, having high −log_10_(*p*) values of 12.11 in 2020 and 9.67 in 2021 (Figure 4). This marker was found in proximity to *Tsn1*. The MLM model identified 19 significant SNP markers at a *p*‐value threshold of 0.001 (Figure 4). Among them, two SNP markers (5A_TA003225‐0566 and 5A_TA003225‐1427) were significant in both years and mean, two (3B_wsnp_Ex_c2723_5047696 and 7B_AX‐94496436) in 2020 and mean, 10 in 2021 and mean, and three (1A_Kukri_c53398_200, 5B_AX‐94730987, and 7D_AX‐95010121) in 2020 only.

### 3.4 Candidate genes for marker‐trait associations

The significant SNPs identified from the GWAS analysis were further studied for the known candidate genes relevant to disease resistance using the recently annotated wheat reference sequence (RefSeq V1.0). Numerous plant protein families encoding proteins with disease‐resistance‐associated domains, such as LRR, PKS, and the MADS transcription factor, were identified in the genomic regions harboring the significant SNPs. The SNP 3A_BS00023028_51 is located within the genomic region 3A:721341896‐721361525 that contains two genes, TraesCS3A02G495300 and TraesCS3A02G495400, which encode for NAD kinase/diacylglycerol kinase‐like domain and leucine‐rich repeat protein kinase‐like domain, respectively.

Similarly, 3B_wsnp_Ex_c2723_5047696 is within the genomic region 3B:6637372‐6682983 that encompasses transcript IDs, namely, TraesCS3B02G016100, TraesCS3B02G016300, TraesCS3B02G016400, and TraesCS3B02G016500 encoding proteins having domains associated with disease resistance (Table 3). SNPs TA003225‐0566 and TA003225‐1427 belong to the genomic region 5A:414406049‐414414131, harboring TraesCS5A02G204800 that encodes ubiquitin‐like domain, UBX domain, and Rad18‐type zinc finger. The SNP 6D_AX‐94499500 is located on the genomic region 6D:10905028‐10947752 having transcript IDs TraesCS6D02G028200 and TraesCS6D02G028300 encoding for disease resistance proteins and associated domains (Table 3).

**TABLE 3:** Single‐nucleotide polymorphisms (SNPs) associated with spot blotch (SB) resistance and the candidate genes.

Sr. No.	SNP	Chromosome	Position	*p* value	Marker *r* ^2^	Experiment	Model	Transcript ID	Function
1	Kukri_c53398_200	1A	47585208	6E‐04	0.074	sb20	MLM	TraesCS1A02G065300	IPR015943 WD40/YVTN repeat‐like‐containing domain
2	BobWhite_c2844_569	1B	636753053	7E‐04	0.073	sbm	MLM	TraesCS1B02G409300	IPR001041:2Fe‐2S ferredoxin‐type iron‐sulfur binding domain
3	Kukri_c59960_211	2D	455333875	4E‐04	0.082	sb21 and sbm	MLM	TraesCS2D02G354400	Aminoacyl‐tRNA synthetase, class I, conserved site
4	BS00023028_51	3A	721354597	7E‐04	0.073	sb21 and sbm	MLM	TraesCS3A02G495300	IPR016064: NAD kinase/diacylglycerol kinase‐like domain superfamily
								TraesCS3A02G495400	IPR001611 Leucine‐rich repeat, IPR011009 protein kinase‐like domain
5	wsnp_Ex_c2723_5047696	3B	6680096	3E‐04	0.082	sb20 and sbm	MLM	TraesCS3B02G016100	IPR045087: Multicopper oxidase
								TraesCS3B02G016300	IPR000719: Protein kinase domain; IPR008271: serine/threonine‐protein kinase, active site
								TraesCS3B02G016500	A0A341S8E6 Fatty acyl‐CoA reductase
								TraesCS3B02G016400	IPR004255 O‐acyltransferase, WSD1, N‐terminal
6	BS00102646_51	3B	6558682	6E‐04	0.081	sb21 and sbm	MLM	TraesCS3B02G015800	IPR008914: Phosphatidylethanolamine‐binding protein
								TraesCS3B02G016000	IPR026055: Fatty acyl‐CoA reductase
7	AX‐94823297	3D	464126211	6.78E‐04	0.074	sb21 and sbm	MLM	TraesCS3D02G353300	IPR019378: GDP‐fucose protein O‐fucosyl transferase
								TraesCS3D02G353400	IPR002885: Pentatricopeptide repeat IPR011990: Tetratricopeptide‐like helical domain
8	Kukri_c6266_260	5A	607682889	7.45E‐05	0.105	sb21 and sbm	MLM	TraesCS5A02G421600	IPR004159: Putative S‐adenosyl‐l‐methionine‐dependent ethyl transferase
								TraesCS5A02G421500	IPR004159: Putative S‐adenosyl‐l‐methionine‐dependent ethyl transferase
9	TA003225‐0566	5A	414411822	2.48E‐04	0.092	sb20, sb21 and sbm	MLM	TraesCS5A02G204800	IPR029071: Ubiquitin‐like domain, IPR001012: UBX domain IPR006642: Zinc finger, Rad18‐type
10	TA003225‐1427	5A	414410915	6E‐05	0.100	sb20, sb21 and sbm	MLM	TraesCS5A02G204800	IPR029071: Ubiquitin‐like domain, IPR001012: UBX domain IPR006642: Zinc finger, Rad18‐type
11	Kukri_c42466_392	5A	414166539	8E‐04	0.075	sbm	MLM	TraesCS5A02G204600	IPR027410: TCP‐1‐like chaperonin intermediate domain, IPR002423: chaperonin Cpn60/TCP‐1 family, IPR002498: phosphatidylinositol‐4‐phosphate 5‐kinase, core, IPR027409: GroEL‐like apical domain
12	AX‐94730987	5B	706341308	6E‐04	0.077	sb20	MLM	TraesCS5B02G559400	IPR002347: Short‐chain dehydrogenase/reductase SDR IPR036291: NAD(P)‐binding domain
								TraesCS5B02G559500	IPR011012: Longin‐like domain
								TraesCS5B02G559600	IPR002885: Pentatricopeptide repeat IPR011990: Tetratricopeptide‐like helical domain
13	AX‐94435238	5B	5.81E+08	7.79E‐13	0.075	sb20, sb21	Farm CPU	TraesCS5B02G405600.1	IPR044824: protein maintenance of meristems‐like; IPR019557: aminotransferase‐like, plant mobile domain
								TraesCS5B02G405700.1	IPR011009: Protein kinase‐like domain superfamily; IPR008271: serine/threonine‐protein kinase, active site
14	GENE‐4086_659	6A	570924201	5.19E‐04	0.078	sb21 and sbm	MLM	TraesCS6A02G336800	IPR002301: Isoleucine‐tRNA ligase, IPR013155: methionyl/valyl/leucyl/isoleucyl‐tRNA synthetase, anticodon‐binding, IPR014729: Rossmann‐like alpha/beta/alpha sandwich fold, IPR023585: Isoleucine‐tRNA ligase, type 1
								TraesCS6A02G336900	IPR029399:TMEM192 family, weakly interact with FRIGIDA, a determinant of flowering time
15	GENE‐4086_876	6B	641286659	1.66E‐04	0.093	sb21 and sbm	MLM	TraesCS6B02G367200	IPR002301: Isoleucine‐tRNA ligase, IPR013155: methionyl/valyl/leucyl/isoleucyl‐tRNA synthetase, anticodon‐binding
								TraesCS6B02G367300	IPR000433: Zinc finger, ZZ‐type IPR013178: histone acetyltransferase Rtt109/CBP
								TraesCS6B02G367400	IPR029399:TMEM192 family, weakly interact with FRIGIDA, a determinant of flowering time
16	AX‐94708023	6B	21138145	2.16E‐04	0.089	sb21 and sbm	MLM	TraesCS6B02G036100	IPR036047: F‐box‐like domain
17	AX‐94948322	6B	19126416	5.10E‐04	0.086	sb21 and sbm	MLM	TraesCS6B02G032600	IPR036047: F‐box‐like domain
								TraesCS6B02G032700	IPR012462: Peptidase C78, ubiquitin fold modifier‐specific peptidase 1/2
18	AX‐94499500	6D	10907336	3.02E‐04	0.089	sb21 and sbm	MLM	TraesCS6D02G028200	IPR011545: DEAD/DEAH box helicase domain, IPR014756: immunoglobulin E‐set IPR027417:P‐loop containing nucleoside triphosphate hydrolase, IPR036390: winged helix DNA‐binding domain
								TraesCS6D02G028300	IPR044974: Disease resistance protein, plants, IPR038005: virus X resistance protein‐like, coiled‐coil domain, IPR032675: leucine‐rich repeat domain, IPR027417: P‐loop containing nucleoside triphosphate hydrolase
19	AX‐94496436	7B	600704585	1.23E‐04	0.102	sb20 and sbm	MLM	TraesCS7B02G344700	PR001229: Jacalin‐like lectin domain, IPR004265: dirigent protein
20	AX‐95010121	7D	378734	8E‐04	0.073	sb20	MLM	TraesCS7D02G000400	IPR039525:E3 ubiquitin‐protein ligase RNF126‐like, zinc‐ribbon, IPR001841: zinc finger, RING‐type
								TraesCS7D02G000500	IPR019410: Lysine methyltransferase, IPR029063: S‐adenosyl‐l‐methionine‐dependent methyltransferase

Abbreviation: MLM, mixed‐linear model.

### 3.5 Stacking of R alleles

**FIGURE 5:**
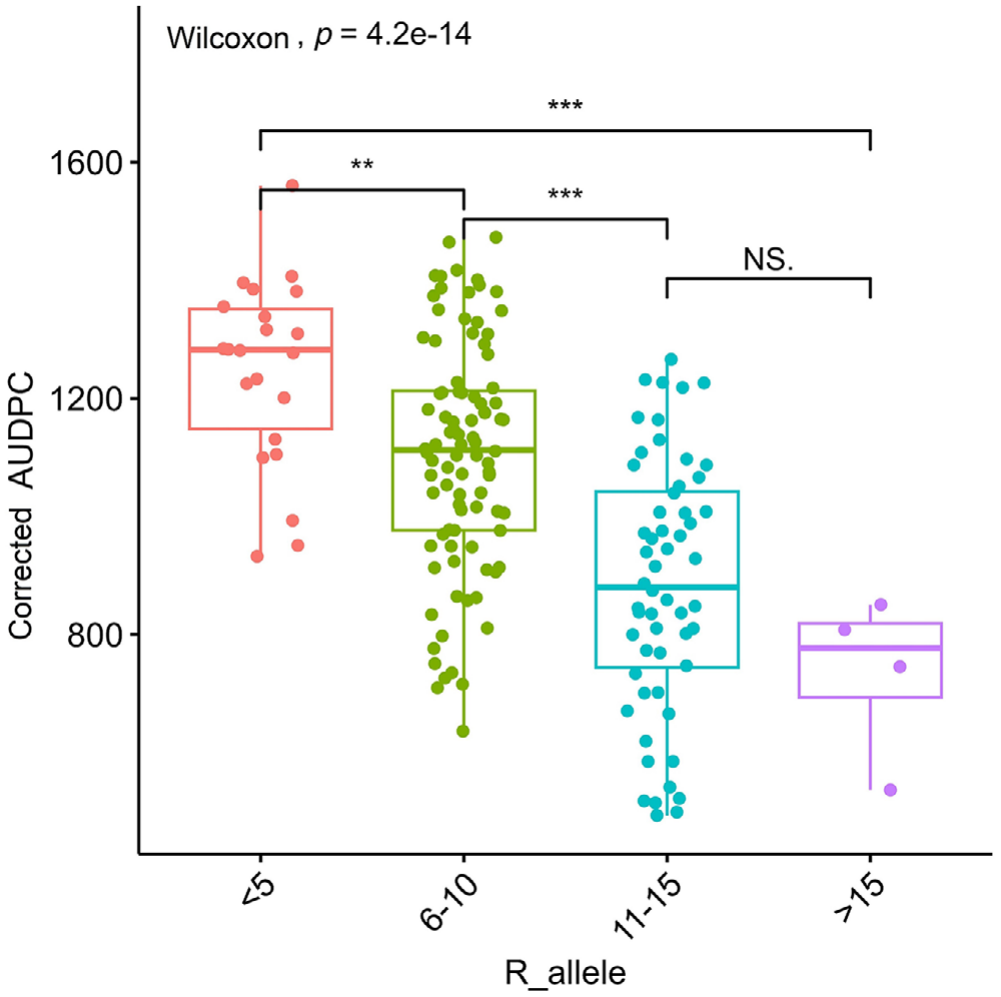
Boxplot for effects of stacking numbers of alleles indicating increased resistance to spot blotch (SB) in the 174 spring wheat lines. AUDPC, area under the disease progress curve. NS, not significant. **p < 0.01. ***p < 0.001.

Nineteen significant markers obtained from the model “MLM” were selected to investigate the effects of pyramiding resistance alleles on SB resistance (Figure 5). Of the 174 lines, SW8488*2/KURUKU (CIM‐87, AUDPC = 536.39) had the highest number (18) of resistance alleles. Three lines, namely, NL1270 (NPL‐20, AUDPC = 850.78), PBW 644 (IND‐7, AUDPC = 808.05), and ATTILA*2/PBW65//KACHU/3/UP2338*2/KKTS*2//YANAC (CIM‐69, AUDPC = 745.41), had 16 resistance alleles. Moreover, 56 genotypes (mean AUDPC = 883.32) had 11–15 resistance alleles, 92 lines (mean AUDPC = 1104.11) had 6–10 resistance alleles, and 22 lines (mean AUDPC = 1247.63) had less than five resistance alleles. The increasing frequency of resistance alleles was associated with a decreasing trend in AUDPC (Figure 5).

### 3.6 Haplotype analysis

**FIGURE 6:**
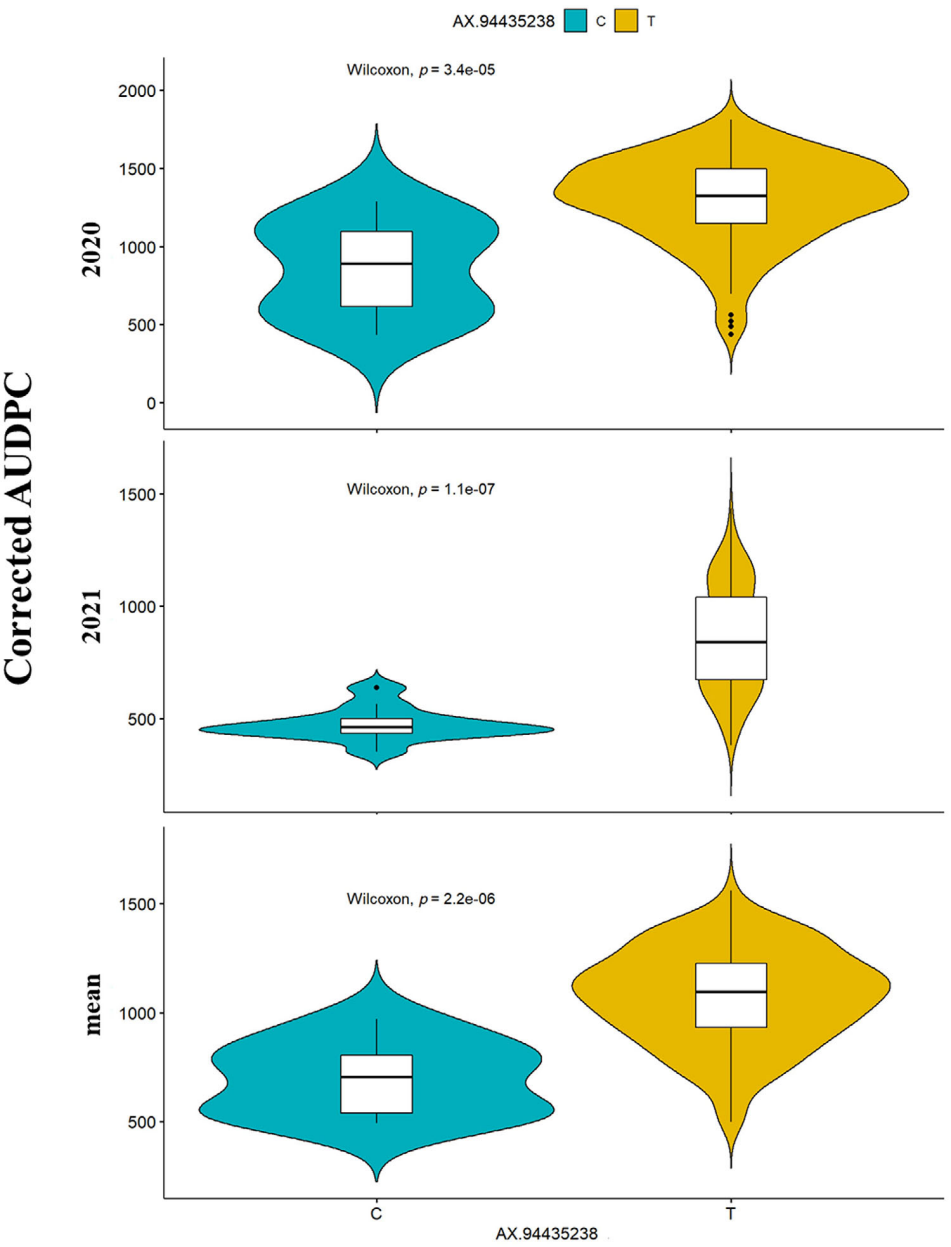
Allelic distribution of significant single‐nucleotide polymorphism (SNP) AX‐94435238 tightly linked to the Tsn1 on chromosome 5B during 2020, 2021 and mean area under the disease progress curve (AUDPC).

**FIGURE 7:**
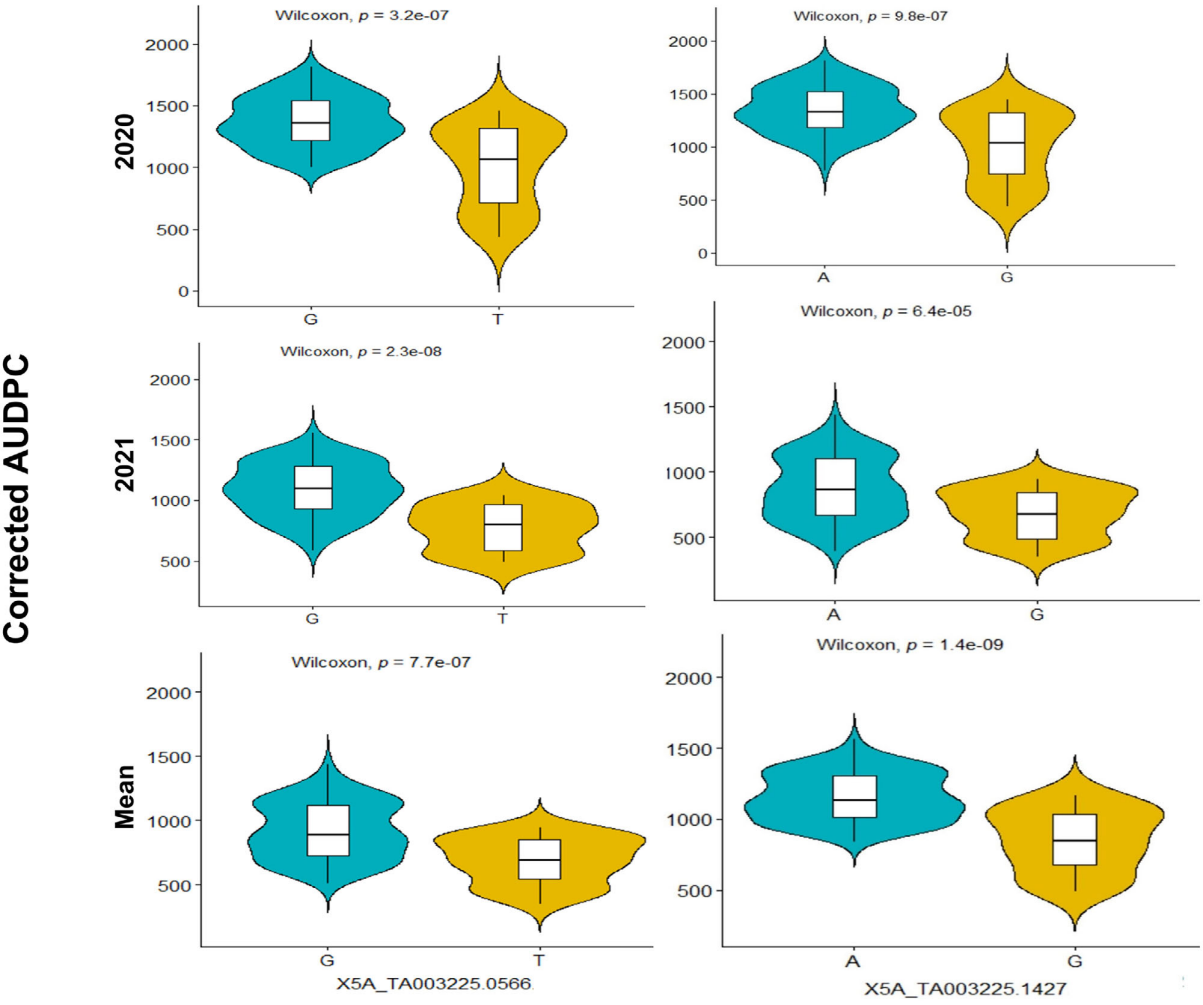
Allelic distribution of significant single‐nucleotide polymorphism (SNP) TA003225.0566 and TA003225.1427 associated with spot blotch (SB) resistance on chromosome 5A.

Haplotype analysis was conducted for three consistently significant SNPs, that is, 5B_AX‐94435238 linked to *Tsn1*, and 5A_TA003225‐0566 and 5A_TA003225‐1427 linked to a possibly new QTL. For 5B_AX‐94435238, the susceptible allele “T” was consistently associated with higher disease severity compared to the resistant allele “C” in both years and the mean (Figure 6). Similarly, the “T” allele of 5A_TA003225‐0566 and the “G” allele of 5A_TA003225‐1427 were consistently associated with SB resistance in the panel (Figure 7).

## 4 DISCUSSION

This study screened a panel of bread wheat genotypes from CIMMYT and South Asia for field SB resistance. Based on the results, several resistant and moderately resistant genotypes were identified, primarily from CIMMYT germplasm. This confirms earlier findings that advanced lines from the CIMMYT bread wheat breeding program possess moderate resistance to SB (Singh et al., 2015). The continuous distribution of disease scores across all experiments indicates the quantitative character of resistance driven by the additive action of numerous QTLs and genes (Ayana et al., 2018; Joshi et al., 2004; Kumar et al., 2007, 2009; Neupane et al., 2007; Singh et al., 2018; Singh et al., 2023). ANOVA revealed significant effects of year and genotype‐by‐year, highlighting the need for multiple evaluations for SB across years/locations to identify lines with stable resistance and reliable markers for breeding (Chattopadhyay et al., 2022; Roy et al., 2021; Singh et al., 2015).

The genetic architecture of resistance to SB in the GWAS panel was proven to be polygenic, and 20 significant SNP markers related to SB resistance were found on 15 chromosomes. The significant marker on 5BL, AX‐94435238, was close to *Tsn1* and likely represented the QTL at this gene locus. The role of *ToxA‐Tsn1* interaction in susceptibility to SB disease has been reported by several researchers (Friesen et al., 2018; McDonald et al., 2018; Navathe et al., 2020), and because the Mexican population of *B. sorokiniana* also carries *ToxA* (Wu et al., 2021), no wonder why this locus turned out to be significant. This underlines the importance of deploying genotypes devoid of *Tsn1* in geographic areas vulnerable to SB. Additionally, because *ToxA* is also widely present in pathogen populations of tan spot and Septoria nodorum blotch, eliminating *Tsn1* from prevalent wheat varieties is also helpful in controlling these two diseases (Navathe et al., 2020).

Apart from AX‐94435238, SNPs 5A_TA003225‐0566 and 5A_TA003225‐1427 were the only consistently detected markers in this study. They were mapped in a chromosomal region 5A:414410915‐414411822, where no QTL for SB resistance has been identified and thus likely represents a new QTL. On the same chromosomal arm, the significant marker 5A_Kukri_c6266_260 was in the genomic region 5A:607678848‐607685776, not very far (20.23Mb) from *Vrn‐A1* that has often been associated with field SB resistance (He et al., 2021; Singh et al., 2018; Zhu et al., 2014); thus, it might be involved in disease escape instead of genetic resistance. Earliness is often associated with SB escape, and a strategy for selecting genotypes based on the early‐flowing allele of *Vrn‐A1* and employing *Sb2* or other SB resistance QTLs to increase SB resistance has been well documented (He et al., 2020; Roy et al., 2021).

The marker Kukri_c59960_211 was located on 2DL at 455333875 bp, with the closest marker for SB resistance being Kukri_c31121_1460 at 607423420 (Ayana et al., 2018), implying that they may represent different QTL. Additionally, the SNP marker 3A_BS00023028_51 in the genomic region 3A:721341896‐721361525 is linked to genes encoding the protein kinase‐like domain, leucine‐rich repeat, and NAD kinase/diacylglycerol kinase superfamily, which is crucial for disease resistance (Tomar et al., 2021).

Two significant markers on 3BS, wsnp_Ex_c2723_5047696 and BS00102646_51, were located in the genomic region 3B: 6532701–6682983, which is different from that reported by Bainsla et al. (2020) at 17.1 Mb but fell into the QTL region for *Sb3* at 6.1–7.1 Mb (Lu et al., 2016). Therefore, these two SNPs likely represent the *Sb3* gene, which was initially reported in a Chinese genotype and recently identified in CIMMYT germplasm (Juliana et al., 2022), highlighting the important role of this gene in conferring SB resistance. The remaining significant SNPs were located within or close to the reported QTLs for SB resistance, for example, 6D_AX‐94499500 was found in the genomic region 6D:10905028‐10947752, which is close to the 6DS QTL reported in Tomar et al. (2021).

This study identified multiple significant SNPs on different chromosomal regions, confirming the quantitative inheritance of SB resistance in the studied panel of wheat genotypes. Nevertheless, a few QTLs on 3BS (at *Sb3*), 5AL, and 5BL (at *Tsn1*) showed relatively big effects and are thus very suitable for marker‐assisted selection (MAS). The effects of stacking resistance alleles confirmed the contribution of minor QTLs in reducing the severity of SB. For example, SW8488*2/KURUKU that possessed the most resistance alleles (18) had a SB severity that was 43% less than genotypes with fewer than five resistance alleles, indicating a clear association between increased number of resistance alleles with decreased SB severity. Using the marker‐assisted backcross method, these QTLs can be transferred into well‐known susceptible varieties or deployed to develop new varieties (He et al., 2022). Genotypes identified in the current study that showed good SB resistance could be utilized in future breeding programmes as resistance donors, and the significant SNP markers could be used to trace their corresponding QTL to speed up the breeding progress.

## AUTHOR CONTRIBUTIONS

**Umesh Kamble**: Conceptualization; data curation; formal analysis; writing—review and editing. **Xinyao He**: conceptualization; data curation; formal analysis; writing—review and editing. **Sudhir Navathe**: Formal analysis; writing—review and editing. **Manjeet Kumar**: Formal analysis; writing—review and editing. **Madhu Patial**: Formal analysis; writing—review and editing. **Muhammad Rezaul Kabir**: Formal analysis; resources; writing—review and editing. **Gyanendra Singh**: Formal analysis; resources; writing—review and editing. **Gyanendra Pratap Singh**: Formal analysis; resources; writing—review and editing. **Arun Kumar Joshi**: Conceptualization; formal analysis; funding acquisition; project administration; resources; supervision; writing—review and editing. **Pawan Kumar Singh**: Conceptualization; data curation; formal analysis; funding acquisition; project administration; resources; supervision; writing—review and editing.

## CONFLICT OF INTEREST STATEMENT

The authors declare no conflicts of interest.

## Supporting information




**Supplementary Table 1** Details of the panel of 174 spring wheat genotypes and their disease response.

## Data Availability

All data supporting the findings of this study are available within the paper and within its Supporting Information.
